# A proteogenomic profile of early lung adenocarcinomas by protein co-expression network and genomic alteration analysis

**DOI:** 10.1038/s41598-020-70578-x

**Published:** 2020-08-12

**Authors:** Toshihide Nishimura, Haruhiko Nakamura, Kien Thiam Tan, De-Wei Zhuo, Kiyonaga Fujii, Hirotaka Koizumi, Saeko Naruki, Masayuki Takagi, Naoki Furuya, Yasufumi Kato, Shu-Jen Chen, Harubumi Kato, Hisashi Saji

**Affiliations:** 1 Department of Translational Medicine Informatics, St. Marianna University School of Medicine, Kawasaki, Kanagawa 216-8511 Japan; 2 Department of Chest Surgery, St. Marianna University School of Medicine, Kawasaki, Kanagawa 216-8511 Japan; 3 ACT Genomics Co., LTD., Taipei, 114 Taiwan; 4 Department of Pathology, St. Marianna University Hospital, Kawasaki, Kanagawa 216-8511 Japan; 5 Division of Respiratory Medicine, Department of Internal Medicine, St. Marianna University School of Medicine, Kawasaki, Kanagawa 216-8511 Japan; 6 Department of Thoracic Surgery, Kanto Central Hospital, Tokyo, 158-8531 Japan; 7 Tokyo Medical University, Tokyo, 160-0023 Japan; 8 International University of Health and Welfare, Tokyo, 107-8402 Japan

**Keywords:** Biological techniques, Biophysics, Cancer, Cell biology, Computational biology and bioinformatics, Molecular biology, Stem cells, Biomarkers, Diseases, Molecular medicine, Oncology, Risk factors

## Abstract

The tumourigenesis of early lung adenocarcinomas, including adenocarcinoma in situ (AIS), minimally invasive adenocarcinoma (MIA), and lepidic predominant invasive adenocarcinoma (LPA), remains unclear. This study aimed to capture disease-related molecular networks characterising each subtype and tumorigenesis by assessing 14 lung adenocarcinomas (AIS, five; MIA, five; LPA, four). Protein–protein interaction networks significant to the three subtypes were elucidated by weighted gene co-expression network analysis and pairwise G-statistics based analysis. Pathway enrichment analysis for AIS involved extracellular matrix proteoglycans and neutrophil degranulation pathway relating to tumour growth and angiogenesis. Whereas no direct networks were found for MIA, proteins significant to MIA were involved in oncogenic transformation, epithelial-mesenchymal transition, and detoxification in the lung. LPA was associated with pathways of HSF1-mediated heat shock response regulation, DNA damage repair, cell cycle regulation, and mitosis. Genomic alteration analysis suggested that LPA had both somatic mutations with loss of function and copy number gains more frequent than MIA. Oncogenic drivers were detected in both MIA and LPA, and also LPA had a higher degree of copy number loss than MIA. Our findings may help identifying potential therapeutic targets and developing therapeutic strategies to improve patient outcomes.

## Introduction

Lung adenocarcinoma is the most common histological subtype of non-small-cell lung cancer (NSCLC), and it accounts for the highest prevalence rate^1^. Lung adenocarcinoma is thought to develop from cells in the distal bronchial epithelia, terminal bronchioles, and alveoli including Clara cells and alveolar type I or type II pneumocytes^2^. About 60% of lung adenocarcinoma subtypes have unique protein markers as oncogenic driver mutations such as *EGFR*, *ALK*, *ROS1*, *HER2*, *KRAS*, and *BRAF*^3^. These are crucial biomarkers in molecular targeted therapy. However, around 40% of lung adenocarcinoma subtypes do not show oncogenic driver mutations, and the prognoses of these patients are relatively poor.

Recent advances in chest high-resolution computed tomography have helped to detect small adenocarcinoma nodules at earlier stages^4,5^. In 2011, the International Association for the Study of Lung Cancer, the American Thoracic Society and the European Respiratory Society proposed a new pathological classification of lung adenocarcinoma. This classification introduced the concepts of adenocarcinoma in situ (AIS) and minimally invasive adenocarcinoma (MIA), resulting in the elimination of the term ‘bronchioloalveolar carcinoma’^6^. While both AIS and MIA included tumours of 3 cm or smaller in size, more specifically, AIS was defined as a pre-invasive lesion showing pure lepidic growth without invasion, whereas MIA was specifically defined as a solitary tumour that also exhibited lepidic predominant growth but showing invasion of 5 mm or less. Conversely, lepidic predominant invasive adenocarcinoma (LPA) with more than 5 mm of invasion is categorised as an invasive adenocarcinoma according to the predominant histological pattern, which can either be acinar, papillary (PAP), micropapillary (MP), solid SLD, or a combination of the four variants.

Lepidic-type adenocarcinomas have been thought to encompass a stepwise progression from AIS to MIA to LPA. Generally, complete AIS or MIA resection leads to 100% recurrence-free 5-year survival^7^. However, some previous studies have reported that LPA might recur after complete resection^8–10^. AIS plus MIA and LPA have different prognoses after resection, and differential protein expressions are thought to be associated with cancer cell invasiveness in each subtype and play important roles in local recurrence and prognosis.

The proteogenomic analysis may be effective in understanding the carcinogenesis and tumorigenesis of each lung adenocarcinoma subtype and identifying specific protein markers to drive differential diagnosis and subtype-specific treatment. Therefore, it is crucial to understand the molecular and tumourigenesis profiles of early lung adenocarcinomas from the perspective of interactions and alterations of disease-related molecular networks.

Recent advances in mass spectrometry (MS) have made MS-based shotgun sequencing and quantitative analysis highly capable of identifying a large number of disease-related proteins expressed in clinical specimens^11–14^. The use of laser microdissection (LMD) enables the collection of a certain type of target cells from formalin-fixed, paraffin-embedded (FFPE) cancer tissue sections. Remarkable advances have been made in next-generation sequencing (NGS), thus enabling complete massive parallel sequencing using only a small number of FFPE specimens to collect comprehensive information regarding copy number variations (CNVs) and mutations in cancer-related genes^15,16^.

Weighted gene co-expression network analysis (WGCNA)^17^ is an extensively applied, unsupervised gene-clustering method designed based on the correlation network of gene expressions^14,18,19^. We have recently identified the key protein modules that characterise small-cell lung carcinoma and large-cell neuroendocrine lung carcinoma using WGCNA and the systematic network analysis of clinical tissue proteome datasets^14^.

Therefore, the present study aimed to identify key protein–protein interaction (PPI) networks that characterise each lung adenocarcinoma subtype and to attain genomic alteration information, which would reflect the disease nature and treatment outcomes in patients with lung adenocarcinoma.

## Results

### Proteome datasets of lung adenocarcinoma

MS-based proteomic analysis was conducted on 14 FFPE tissue specimens of lung adenocarcinomas: five AIS, five MIA, and four LPA. These specimens were selected for their preserved condition, tumour area, and well-clarified pathological diagnosis (Table 1). Pre-surgical treatment was not performed for any of these lung adenocarcinomas.

**Table 1:** Clinicopathological information of patients with early lung adenocarcinoma.

Sample no	Histological type	Age (years)	Sex	Location	Tumour size on CT (mm)	Clinical TNM classification			Clinical stage	*EGFR* mutation
						c-T	c-N	c-M		
**A. AIS (n = 5)**										
AIS_T53	AIS	53	F	RS8	11	cT1a	cN0	cM0	cIA	*EGFR*( +)
AIS_T54	AIS	74	M	RS6	29	cT1b	cN0	cM0	cIA	*EGFR*( −)
AIS_T56	AIS	69	F	RS6	16	cT1a	cN0	cM0	cIA	*EGFR*( −)
AIS_T58	AIS	78	M	LS1 + 2	28	cT1b	cN0	cM0	cIA	*EGFR*( −)
AIS_T59	AIS	68	F	RS1	13	cT1a	cN0	cM0	cIA	*EGFR*( +)
	Average ± SD	68.4 ± 8.5			19.4 ± 7.6					
**B. MIA (n = 5)**										
MIA_T73	MIA	58	M	RS9	20	cT1a	cN0	cM0	cIA	*EGFR*( +)
MIA_T74	MIA	67	F	LS3	19	cT1a	cN0	cM0	cIA	*EGFR*( +)
MIA_T75	MIA	77	F	RS8	20	cT1a	cN0	cM0	cIA	*EGFR*( −)
MIA_T79	MIA	61	M	LS6	10	cT1a	cN0	cM0	cIA	*EGFR*( +)
MIA_T80	MIA	63	F	RS3	12	cT1a	cN0	cM0	cIA	*EGFR*( −)
	Average ± SD	65.2 ± 6.6			16.2 ± 4.3					
**C. LPA (n = 4)**										
LPA_T85	LPA	68	M	RS1	30	cT1b	cN0	cM0	cIA	*EGFR*( −)
LPA_T87	LPA	73	F	RS3	28	cT1b	cN0	cM0	cIA	*EGFR*( +)
LPA_T88	LPA	59	M	LS1 + 2	30	cT1b	cN0	cM0	cIA	*EGFR*( −)
LPA_T89	LPA	67	F	RS6	20	cT1a	cN0	cM0	cIA	*EGFR*( −)
	Average ± SD	66.8 ± 5.0			27.0 ± 4.1					
*p* value_ANOVA		0.816			0.077					

AIS, adenocarcinoma in situ; MIA, minimally invasive adenocarcinoma; LPA, lepidic predominant invasive adenocarcinoma; ADC, adenocarcinoma; ANOVA, analysis of variance.

**Figure 1:**
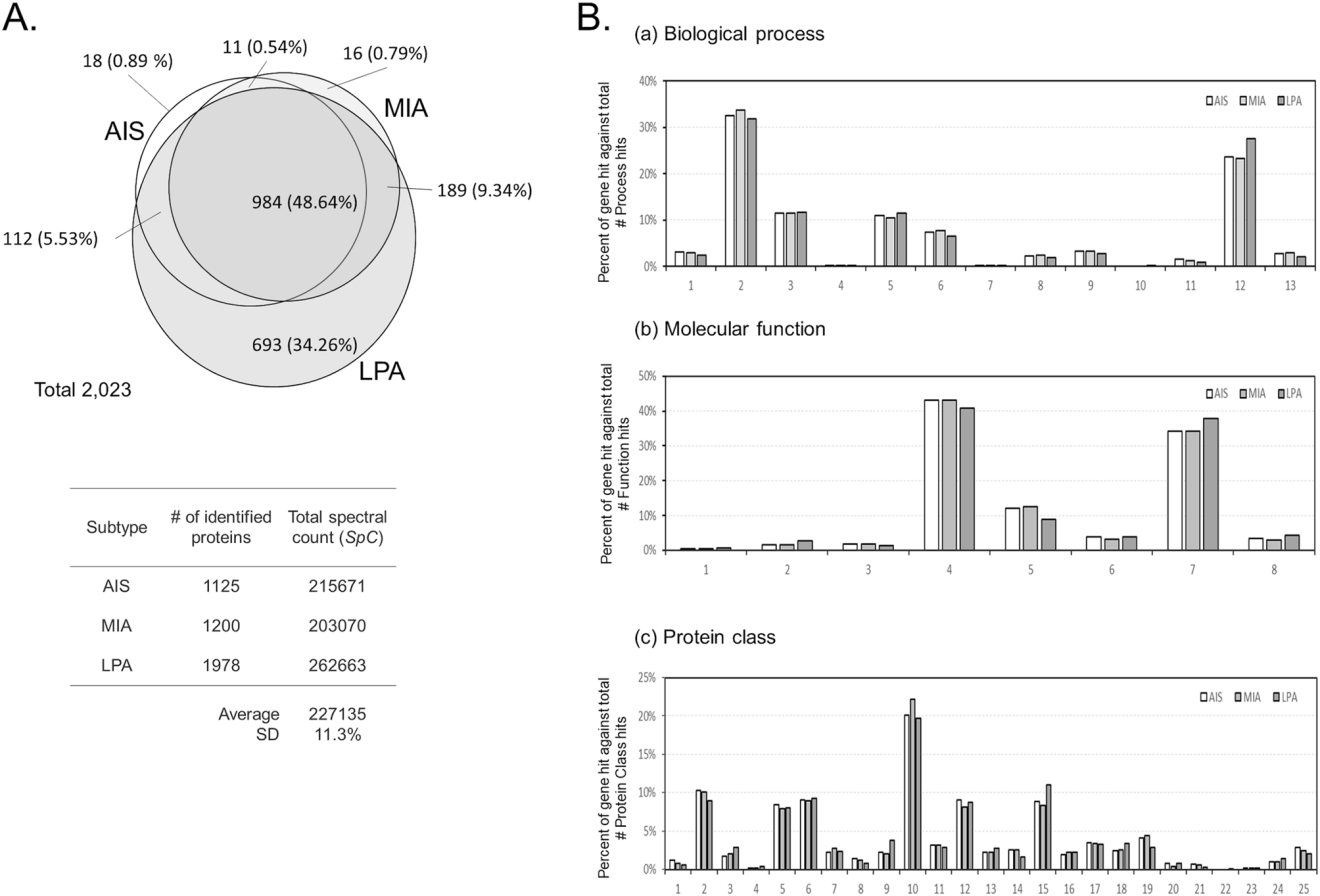
Venn map and hierarchical clustering of the identified proteins. (**A**) Venn map of the identified proteins. (**B**) Gene ontology (GO) analysis of the identified proteins to AIS, MIA and LPA. (a) Biological process. 1, cellular component organization or biogenesis (GO:0071840); 2, cellular process (GO:0009987); 3, localization (GO:0051179); 4, reproduction (GO:0000003); 5, biological regulation (GO:0065007); 6, response to stimulus (GO:0050896); 7, pigmentation (GO:0043473); 8, developmental process (GO:0032502); 9, multicellular organismal process (GO:0032501); 10, rhythmic process (GO:0048511); 11, biological adhesion (GO:0022610); 12, metabolic process (GO:0008152); 13, immune system process (GO:0002376). (b) Molecular function. 1, translation regulator activity (GO:0045182); 2, transcription regulator activity (GO:0140110); 3, molecular transducer activity (GO:0060089); 4, binding (GO:0005488); 5, structural molecule activity (GO:0005198); 6, molecular function regulator (GO:0098772); 7, catalytic activity (GO:0003824); 8, transporter activity (GO:0005215). (c) Protein class. 1, extracellular matrix protein (PC00102); 2, cytoskeletal protein (PC00085); 3, transporter (PC00227); 4, transmembrane receptor regulatory/adaptor protein (PC00226); 5, transferase (PC00220); 6, oxidoreductase (PC00176); 7, lyase (PC00144); 8, cell adhesion molecule (PC00069); 9, ligase (PC00142); 10, nucleic acid binding (PC00171); 11, signaling molecule (PC00207); 12, enzyme modulator (PC00095); 13, calcium-binding protein (PC00060); 14, defense/immunity protein (PC00090); 15, hydrolase (PC00121); 16, transfer/carrier protein (PC00219); 17, membrane traffic protein (PC00150); 18, transcription factor (PC00218); 19, chaperone (PC00072); 20, cell junction protein (PC00070); 21, surfactant (PC00212); 22, structural protein (PC00211); 23, storage protein (PC00210); 24, isomerase (PC00135); 25, receptor (PC00197).

A total of 2,023 proteins were identified; among these, about 48.6% were commonly expressed in the cancer cells of the three aforementioned subtypes of lung adenocarcinoma (Fig. 1). Overall, < 1% of unique to AIS and MIA each, whereas ca. 34% of the identified proteins were unique to LPA (Fig. 1A).

When a protein is expressed with its spectral count (*SpC*) ≥ 0 and in more than three samples of a subtype, the protein can be referred to be characteristic to the lung adenocarcinoma subtype. The spectral count (*SpC*) is the number of tandem MS/MS spectra assigned to each protein. Overall, 760, 673, and 1,357 proteins were characteristic to AIS, MIA, and LPA, respectively. The gene ontology (GO) analysis was performed using the Protein Analysis Through Evolutionary Relationships (PANTHER) version 14.1 software program (Paul D. Thomas, University of Southern California, Los Angeles, CA, USA)^20^, and presented its results quite similar among the three subtypes (Fig. 1B). Commonly to all three subtypes, proteins were abundantly associated with cellular process, localization, biological regulation, response to stimulus and metabolic process in biological process (GO), binding, structural molecule activity and catalytic activity in molecular function (GO), and cytoskeletal protein, transferase, oxidoreductase, nucleic acid binding, enzyme modulator, and hydrolase in protein class (GO) (Fig. 1B).

### Identification of key protein modules by WGCNA

**Figure 2:**
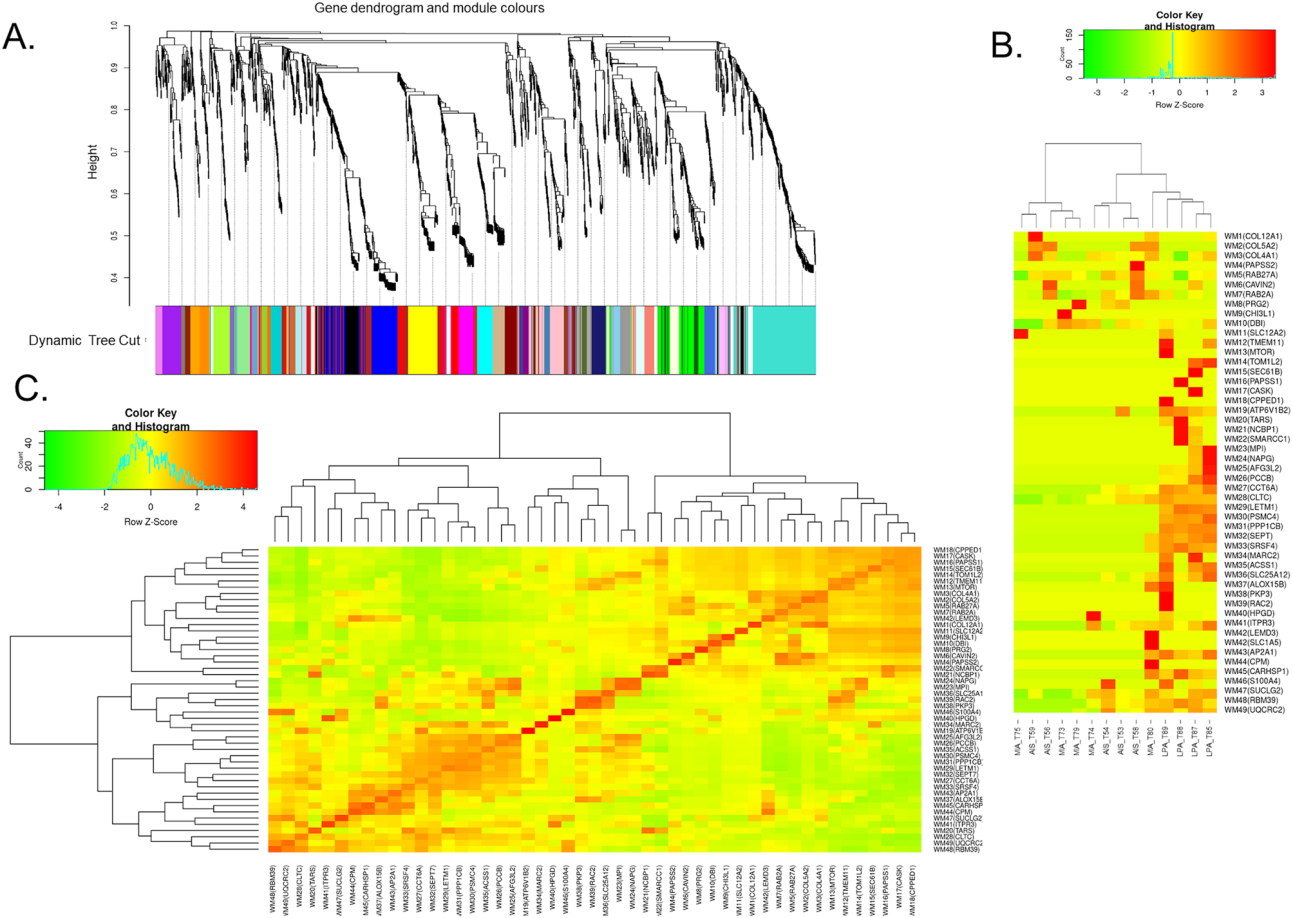
Gene modules identified by WGCNA. (**A**) Gene dendrogram obtained by clustering dissimilarity according to topological overlap with the corresponding module. The coloured rows correspond with the 49 modules identified by dissimilarity according to topological overlap. (**B**) A heatmap of the proteome abundance of eigen proteins in the 49 protein modules and samples. (**C**) Pairwise correlations between the modules in the heatmap of eigen-protein expressions.

We constructed a weighted gene co-expression network and clustered all the identified proteins, and identified 49 protein modules (Fig. 2A). In the WGCNA, a soft threshold power of 10 was selected to define the adjacency matrix according to the criteria of approximate scale-free topology, with a minimum module size of 10 and a module detection sensitivity *deepSplit* of 4. The clinical traits for patients were set according to the lung adenocarcinoma subtype—AIS, MIA, or LPA. The correlations between the resultant modules and clinical traits were determined to identify the protein modules whose expressions were upregulated or downregulated in the subtype samples. A heatmap of the proteome abundance of eigen-proteins and samples and pairwise correlations between the modules in the heatmap of eigen-protein expressions were presented (Fig. 2B, C).

**Figure 3:**
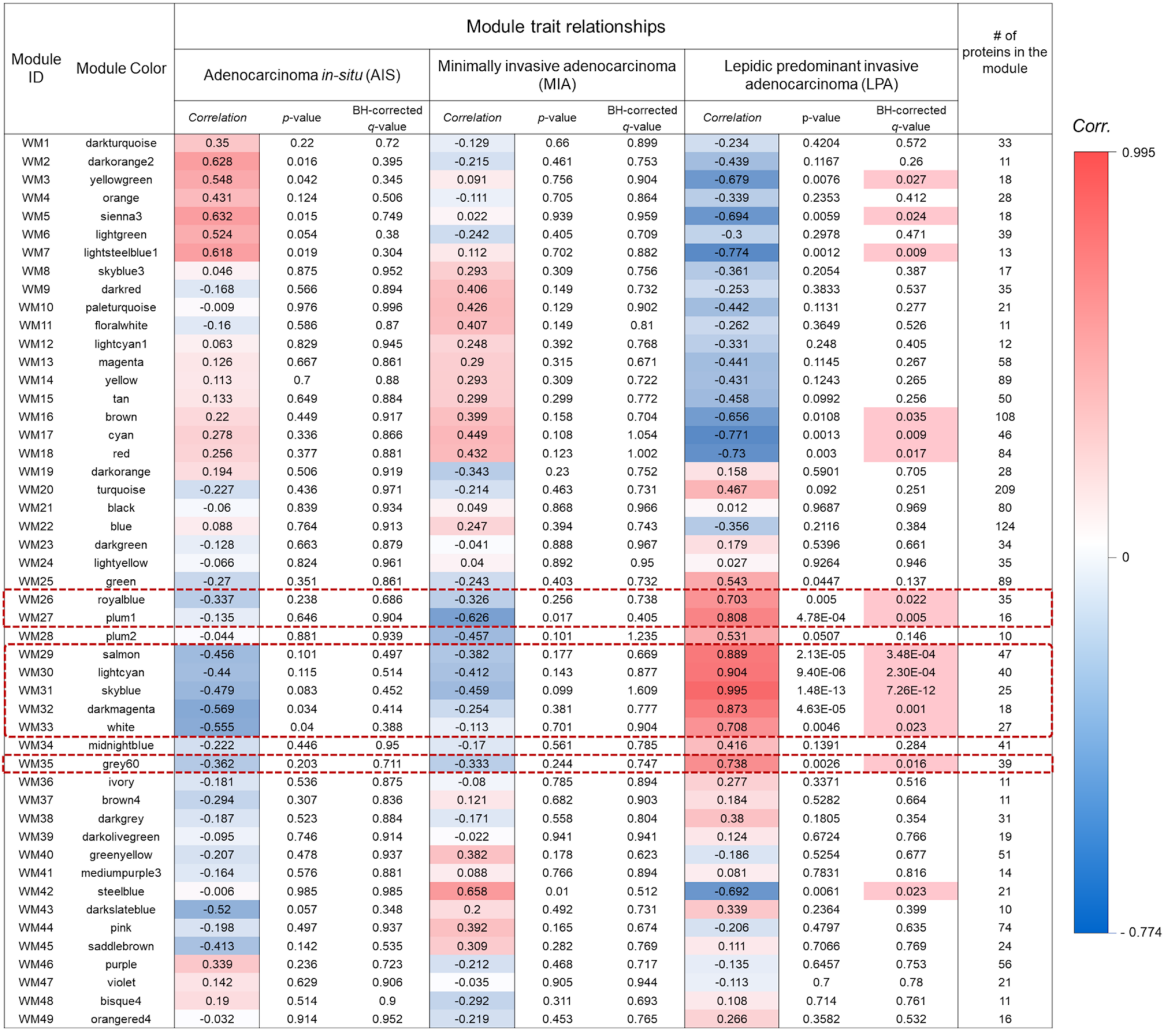
Relationship between module eigen-proteins and the clinical traits of subtypes AIS, MIA, and LPA. Each row in the embedded table represents weighted gene co-expression network analysis results for each module. The first and second columns in the table represent the module identification and colour name of the module, respectively. The twelfth column represents the number of proteins in each module. The *p* values of the correlation coefficients and *q* values by multiple testing correction using the Benjamini–Hochberg method are presented. The table is colour-coded by the correlation coefficient according to the colour legend on the right side of the figure. The intensity and direction of the correlations are indicated on the right side of the heatmap (red, positive correlation; blue, negative correlation). Columns with significant *q *values are highlighted in bright red background.

We identified several modules that showed high and/or moderate correlations with clinical traits (correlation: |r|> 0.5) (Fig. 3). We performed multiple testing correction by the Benjamini–Hochberg method. Finally, the eight modules—WM26, WM27, WM 29, WM30, WM31, WM32, WM33, and WM35 (indicated by the red dashed squares) associated with LPA were found to be statistically significant whereas none of the modules associated with AIS or MIA remained significant.

### Identification of the PPI networks associated with AIS and MIA by pairwise G-statistical analysis of identified proteins

**Figure 4:**
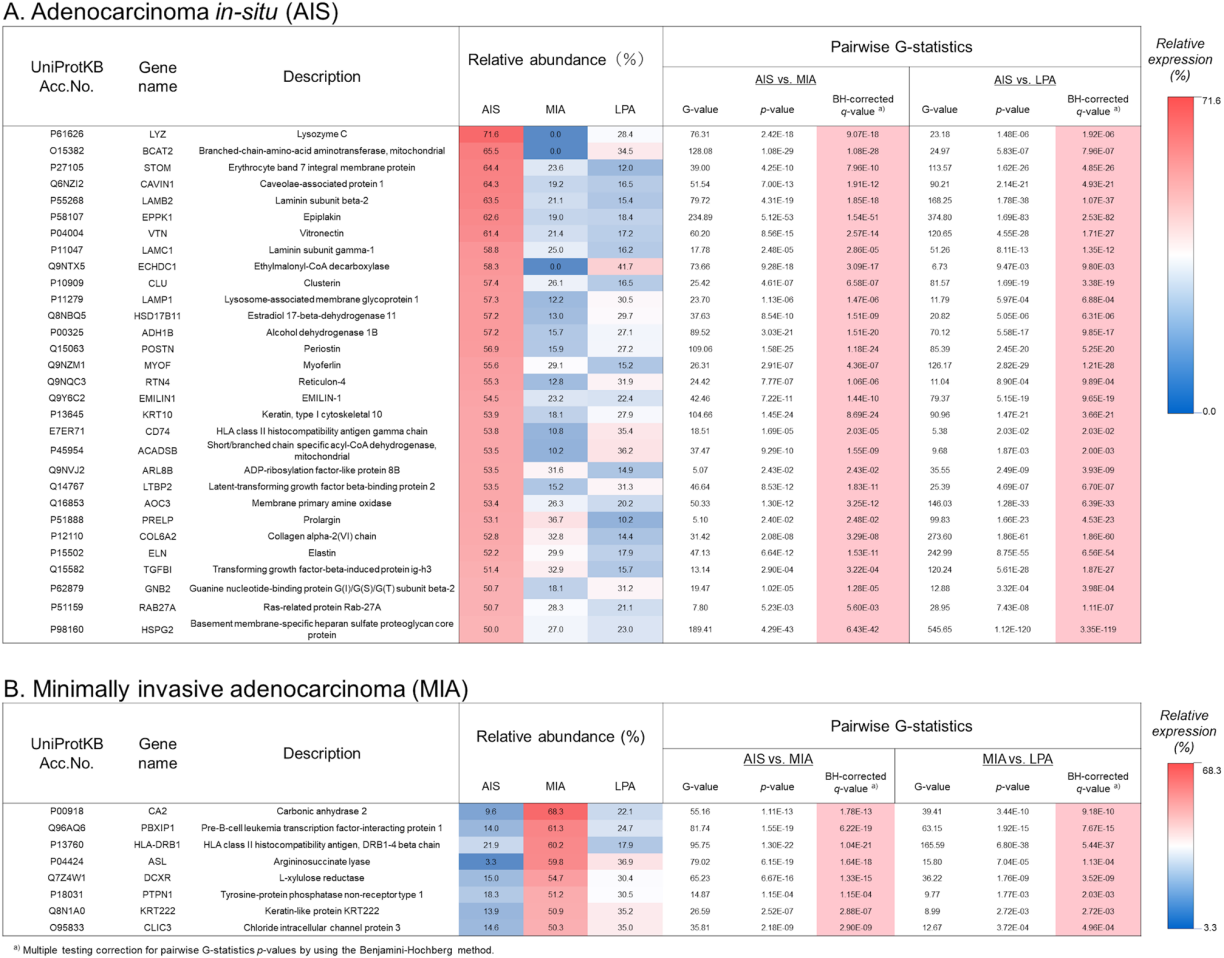
Proteins significant to AIS and MIA identified by pairwise G-statistics analysis^21^. The *q* values by multiple testing correction using the Benjamini–Hochberg method are presented.

Trait analysis in WGCNA trends to overlook important modules for investigating differential disease mechanisms whereas WGCNA is a powerful tool in identifying the co-expression of molecular modules. Indeed, we could not identify key co-expression modules associated with AIS and MIA with high statistical confidence. We adopted the pairwise G-statistics approach^21^ that can identify individual proteins with significant differences in spectral counting (*SpC*s)-based proteome abundance among different patient groups, where William’s correction for continuity was applied to the 2 × 2 tables^13^. The adjusted G-statistical calculation enabled us to handle the data containing small spectral counts including zero. A protein was defined to be significant to AIS or MIA when the protein was expressed in ≥ 60% samples of the group and has its relative abundance > 50% and *q* values < 0.05 that were corrected by the Benjamini–Hochberg method for pairwise G-statistics *p* values among the three groups. We thus identified thirty and eight proteins significant to AIS and MIA, respectively (Fig. 4).

### Functional enrichment analysis of the PPI networks

**Figure 5:**
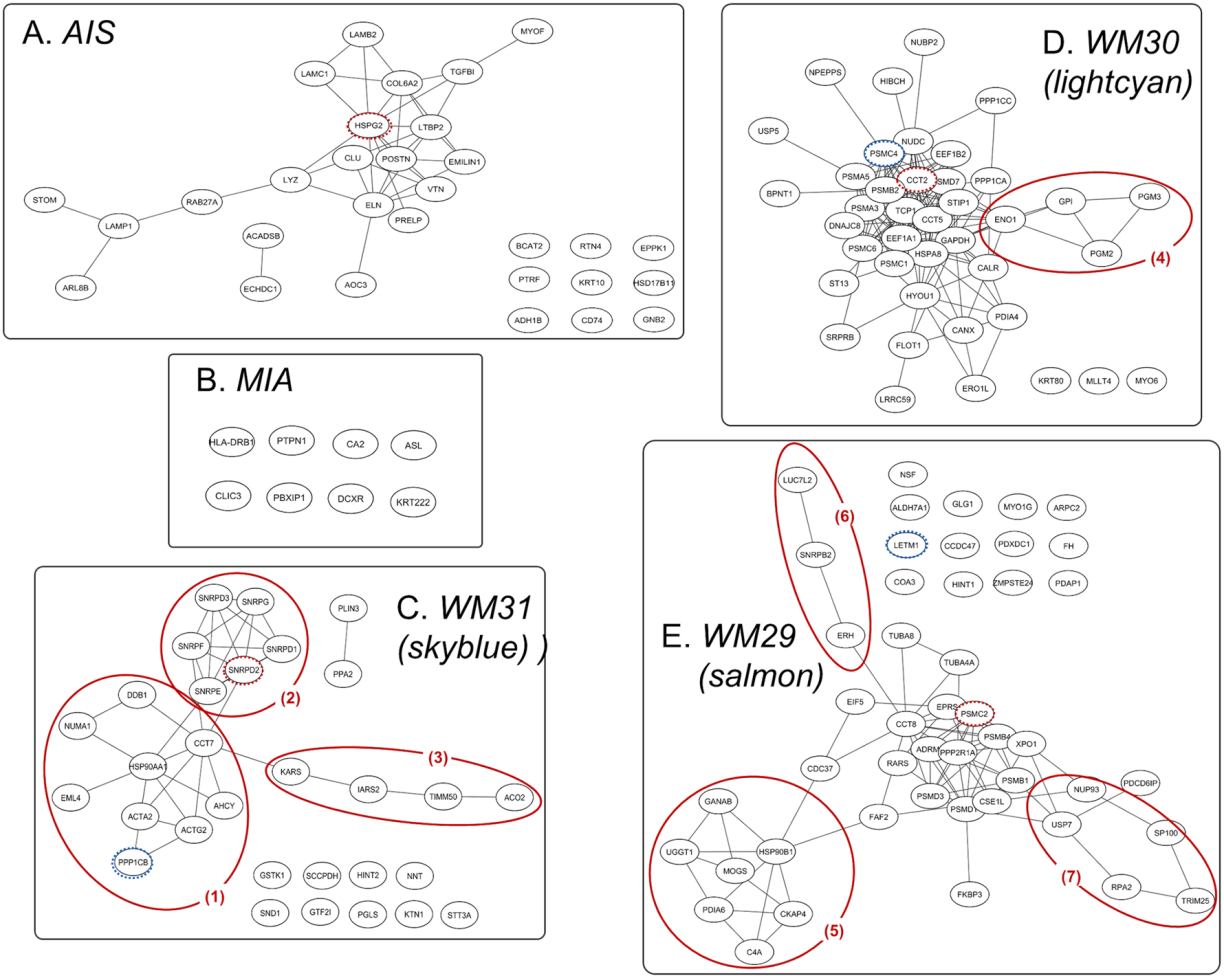
Protein–protein interaction networks identified for (**A**) AIS and (**B**) MIA, and the top three LPA protein networks: (**C**) WM31, (**D**) WM30, and (**E**) WM29 modules. Dotted circle nodes in blue and red represent eigen-proteins and hub proteins, respectively, for each module. Solid red circles with numbers represent subnetworks.

**Figure 6:**
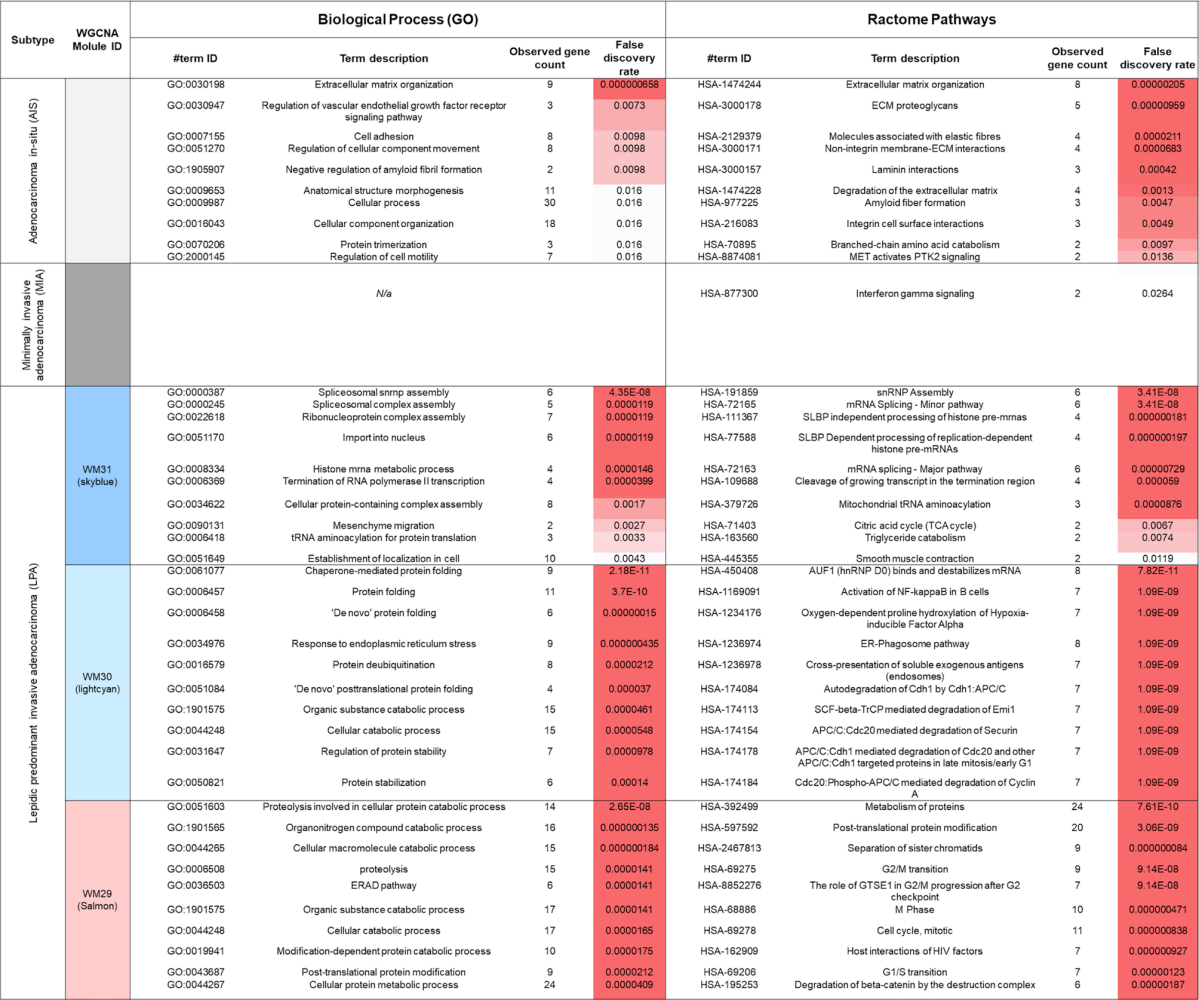
Top pathways enriched for the protein core networks obtained for AIS, MIA, and LPA concerning Biological Process (GO) and Reactome pathways.

To characterise the key protein networks, the biological association among the proteins in each network was analysed by mapping the network proteins in the human protein–protein interaction (PPI) network and among the biological pathways by pathway enrichment analysis (Figs. 5 and 6).

The PPI networks generated using the Search Tool for the Retrieval of Interacting Genes/Proteins (STRING) database^22^ were reconstructed using the CYTOSCAPE version 3.7.2 software program (Institute for Systems Biology, Seattle, WA, USA) for the protein networks of AIS and MIA identified by the pairwise G-statistics and the eight WGCNA network modules associated with LPA (WM26, WM27, WM 29, WM30, WM31, WM32, WM33, and WM35). Top hub proteins were identified according to maximal clique centrality (MCC) by using in the CYTOHUBBA PLUGIN^23^. Figure 5 shows the protein networks of AIS, MIA, and the top three WGCNA modules—WM 29, WM30 and WM31 highly correlated with LPA, where eigen- and/or hub-proteins in their networks were indicated by blue and red dotted circles, respectively. Figures S1 and S2 in Supplementary Information File 1 represent core protein networks and their pathways enriched for WM26, WM27, WM32, WM33, and WM35.

The enriched pathways of the protein networks associated with AIS (Fig. 5A) included (1) biological process (GO): extracellular matrix organisation, regulation of vascular endothelial growth factor receptor (VEGFR) signalling pathway, and cell adhesion; (2) Reactome pathways: extracellular matrix organization, ECM proteoglycans, molecules associated with elastic fibres, and non-integrin membrane-ECM interactions. The top MCC-hub protein heparan sulfate proteoglycan 2 (HSPG2) (also known as Perlecan) is an integral component of basement membranes and interacts with other ECM components as captured in the PPI networks associated with AIS involving laminin (LAMB2 and LAMC1) and prolargin (PRELP) (Fig. 5A). Perlecan binds growth factors via heparan sulfate chains and interacts with vascular endothelial growth factor receptors 2 (VGFR2) which plays a major role in tumour angiogenesis. Dynamic remodelling of tissue architecture takes place during tumour growth and angiogenesis, where perlecan is one of the ECM constituents of the tumour microenvironment. Whereas the intact protein perlecan is known to possess pro-angiogenic properties, its C-terminal fragment, which is released by proteolysis during cancer remodelling and known as endorepellin, has the opposite functions of anti-angiogenic activity^24^. Perlecan interacts with elastin (ELN), vitronectin (VTN), LTBP2, TGFBI, elastin microfibril interfacer 1 (EMILIN1), collagen alpha-2(VI) chain (COL6A2), periostin (POSTN), and lysozyme C (LZY) (as seen in Fig. 5A) which are involved in ECM organization and cell adhesion. The subnetwork of STOM, LAMP1, ARL8B, and RAB27A, and LYZ (Fig. 5A) suggests an involvement of the neutrophil degranulation pathway. Recently, the role of the neutrophil degranulation has been discussed how the release of neutrophil granule proteins is associated with cancer development and tumour progression via neutrophil-mediated transport of cancer cells leading to different cellular phenotypes and into different tissues^25^. The subnetwork consisting of ACADSB and ECHDC1 may relate to the carboxylic acid catabolic process.

No protein networks were obtained in-network depth of 0 interactions mainly because of the limited number of proteins significant to MIA by which no hub proteins were identified (Fig. 5B). The only enriched pathway was obtained as interferon-gamma signalling in Reactome pathways (Fig. 6), which included MHC class II HLA-DR beta 1 chain (HLA-DRB1) and protein tyrosine phosphatase non-receptor type 1 (PTPN1). PTPN1 (also known as PTP1B) belongs to protein tyrosine phosphatases (PTPs) family. PTP1B has been known to have the two faces in tumorigenesis that PTP1B promotes tumour progression in some cancers but functions as a tumour suppressor in other cancers^26^, whereas the role of PTP1B in NSCLC has been unknown. It has been reported that the high expression of PTP1B in NSCLC tissues was associated with the stage and overall survival of NSCLC patients^26^. PTP1B inhibitors are currently considered a promising anti-cancer therapy due to its involvement in the progression of numerous types of cancers via oncogenic transformation^27^. Hematopoietic pre-B-cell leukemia transcription factor (PBX)-interacting protein (PBXIP1), also known as HPIP, is a corepressor of PBX. Shi et al.^28^ demonstrated that HPIP silencing suppressed TGF-β1-induced epithelial-mesenchymal transition (EMT) in A549 lung cancer cells in vitro. DCXR encodes diacetyl/l-xylulose reductase, a multifunctional enzyme in glucose metabolism. Reactive carbonyls are known to cause severe respiratory diseases, which are detoxified by carbonyl reductases in the lung, in particular, DCXR that mediates chemical redox cycling^29^.

The eight WGCNA modules significant to LPA (Fig. 5C–E and Fig. S1A–E in Supplementary Information File 1) were subjected to further bioinformatics analysis. The top WGCNA modules were WM31, WM30, and WM29 in the order of correlation values: 0.995, 0.904, and 0.889, respectively. The enriched pathways of the WM31 (Fig. 5C) included (1) biological process (GO): spliceosomal snRNP assembly and termination of RNA polymerase II transcription; (2) Reactome pathways: snRNP assembly, mRNA splicing—minor pathway, histone stem-loop-binding protein (SLBP) independent processing of histone pre-mRNAs, cleavage of a growing transcript in the termination region, and citric acid cycle (TCA cycle). The eigengene *PPP1CB* encodes serine/threonine-protein phosphatase 1 (PP1)-beta catalytic subunit (PP-1B) which is one of the catalytic subunits of PP1 involved in regulating cell division. Low levels of PP1s and their key regulatory subunit PPP1R9B (known as Spinophilin) are related to a poor prognosis in numerous cancers including lung cancers, which was more involved in squamous cell lung carcinoma than lung adenocarcinoma^30^. It might be noteworthy that a malignant glioma of infancy was recently found to harbour a novel PPP1CB-ALK fusion protein^31^, to be compared with the EML4-ALK translocation mutation present in ca. 6% of NSCLC. The main PPI network (1) including PP-1B, damage specific DNA binding protein 1 (DDB1) and nuclear mitotic apparatus protein 1 (NUMA1) (Fig. 5C) is involved in mesenchyme migration, cell cycle, mitotic/recruitment of NuMA to mitotic centrosomes and regulation of Polo-like kinase 1 (PLK1) activity at G2/M Transition. The subnetwork (2) including the hub protein small nuclear ribonucleoprotein Sm D2 (SM-D2) encoded by *SNRPD2* belongs to the spliceosomal snRNP assembly, and the subnetwork (3) including KARS, IARS2, TIMM50, and ACO2 is involved in the tRNA aminoacylation for protein translation and carboxylic acid metabolic process.

The enriched pathways of the WM30 module included (1) biological process (GO): chaperone-mediated protein folding, response to endoplasmic reticulum stress, and protein de-ubiquitination; (2) Reactome pathways: AU-rich element RNA-binding protein 1 (AUF1) (also known as hnRNP D0) binding and destabilisation of mRNA, activation of nuclear factor kappa B (NF-κB) in B-cells, and oxygen-dependent proline hydroxylation of hypoxia-inducible factor-alpha (HIF-alpha) (Figs. 5D and 6). The eigengene *PSMC4* encodes 26S proteasome regulatory subunit 6B, a component of the 26proteasome, playing a key role in the maintenance of protein homeostasis by removing misfolded or damaged proteins. The translation elongation factor eukaryotic elongation factor 1-alpha1 (EEF1A1) was reported to participate in the entire heat shock response process from transcription through translation^32^. Depending on the type of stimulus, EEF1A1 can be phosphorylated and/or methylated and can interact with trans-acting factors including AUF1, which possibly determines the stability of specific mRNAs and its interaction with 70-kd heat shock protein (HSP70s) mRNA. Stress-induced phosphoprotein 1 (STIP1), also known as HOP/P60/STI1, is a chaperone protein that comprises three tetratricopeptide repeat domains, which can simultaneously bind HSP70s and HSP90s. STIP1 is a tumour-associated antigen (TAA)^33^; its overexpression has been identified in numerous cancers, including colorectal carcinoma^34^, pancreatic cancer^35^, cholangiocellular carcinoma^36^ and ovarian cancer^37^, and it is possibly associated with poor survival outcomes in patients with cancer^38^. *ST13* encodes ST13 HSP70s-interacting protein (HIP)/putative tumour suppressor ST13/suppression of tumorigenicity 13 protein, which is also involved in the regulation of heat shock factor 1 (HSF1)-mediated heat shock response and which mediates the association of HSP70s and HSP90s. In an in vitro study on pancreatic ductal adenocarcinoma, Ma et al.^39^ reported that signal recognition particle receptor subunit beta (SRPRB) plays a central role in the interaction between proteins and stress-associated endoplasmic reticulum (ER) protein 1 (SERP1), which is responsible for the accumulation of unfolded protein in ER stress. They also noted that the downregulation of SERP1 significantly increased SRPRB expression, leading to cell apoptosis through NF-κB activation. Hypoxia upregulated protein 1 (HYOU1) has a significant degree of interaction with SRPRB that well correlated with both the upregulated expressions of SRPRB and HYOU1 observed in our protein expression data. Yamaguchi et al.^40^ found that SERP1 expression was enhanced in vitro by hypoxia, which was associated with the accumulation of unfolded protein in ER stress. The subnetwork (4) is involved in the pathway of carbohydrate metabolic process.

### A genomic alteration analysis for MIA and LPA

**Figure 7:**
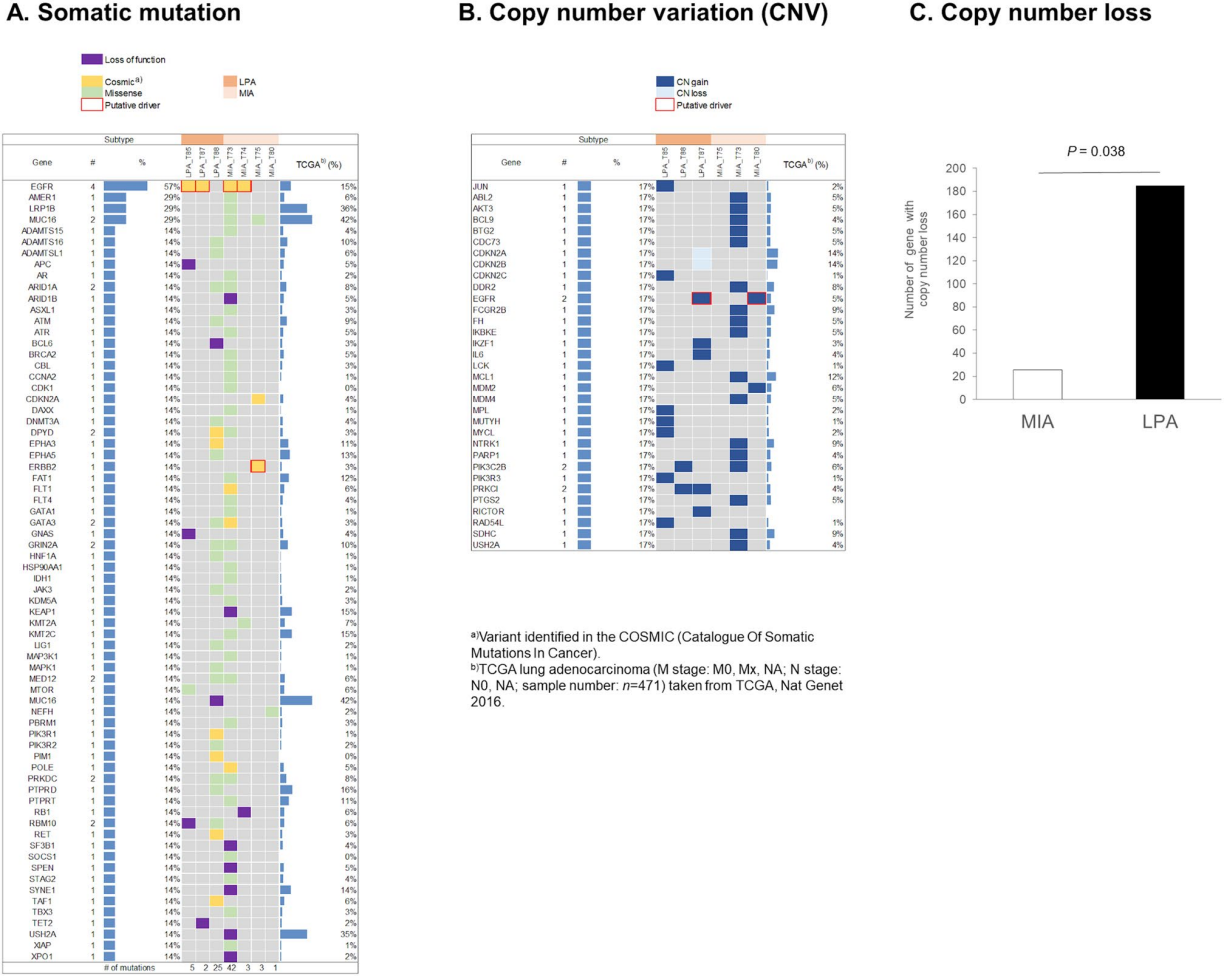
Genomic alterations obtained for MIA and LPA. (**A**) Somatic mutations, and (**B**) copy number variations (CNVs) together with the TCGA lung adenocarcinoma datasets (sample size: *n* = 471). (**C**) Analysis of copy number loss between MIA and LPA. There are significant more genes with copy number loss (CN < 2) found in the LPA group (*p* = 0.038).

Protein expression profiles were found to be highly differentiated between MIA and LPA (Figs. 1A and 2B). We then conducted NGS to obtain genomic alteration profiles in FFPE tissue specimens, which were obtained from the same FFPE blocks used for the proteomic analyses. NGS was performed to identify genomic alterations such as gene mutations including single-nucleotide variations, insertion/deletion variations, and gene CNVs, using the ACTONCO panel (ACT Genomics, Taipei, Taiwan), which comprises of 440 cancer-related genes. Oncoprints were prepared to visualise somatic mutations and CNVs by heatmap together with the TCGA lung adenocarcinoma (ADC) datasets for comparison (Fig. 7A, B). TCGA lung ADC datasets (M stage: M0, Mx, NA; N stage: N0, NA; sample number: *n* = 471) were obtained from the cBioPortal Pan-Lung cancer (TCGA, Nat Genet 2016) (https://www.cbioportal.org/). LPA was suggested to accompany both somatic mutations with loss of function and copy number gains more frequent than the MIA group. Given the small sample size, it is hard to perform statistical analysis or to find any enriched biomarkers in MIA or LPA1. However, it was shown that all both MIAs and LPAs harboured at least one oncogenic driver with EGFR oncogenic mutations being most prevalent. Other oncogenic events detected include ERBB2 p. Gly776delinValVal in the sample MIA_T75, and EGFR copy number gain in the samples of LPA_T87 and MIA_T80. Oncogenic drivers identified in our study were quite similar to those from the large-scale genomic studies^41^. Aside from the tumour which exhibited the APOBEC-like mutation signature, in our cohort, a high percentage of MIAs and LPAs were EGFR mutant, which may explain why the number of somatic mutations is low and no difference in tumour mutational burden was found between two groups.

## Discussion

The tumourigenesis of early lung adenocarcinoma is thought to progress in a stepwise manner in the order of AIS, MIA, and LPA; however, this has not been completely elucidated to date. Therefore, understanding of disease-related molecular mechanisms and profiles in early lung adenocarcinomas would be markedly useful for selecting treatment strategies and could improve the treatment outcomes of individual patients. Protein-based networks by both WGCNA and pairwise G-statistics analyses identified several protein modules and networks that were potentially associated with disease mechanisms driven by distinct early lung adenocarcinomas.

Most activities in ECM organization and integrin-mediated cell–cell adhesion characterised by AIS were consistent with its growth pattern restricted to neoplastic cells with pre-existing alveolar structures without stroma, vascular, or pleural invasion. MIA might be associated with oncogenic transformation and detoxification in lung cell-extracellular matrix interactions although no direct networks were identified. This seems to coincide with the observation of tumour histology defined as MIA presenting a limited size of invasion with a myofibroblastic stroma-associated invasive tumour. Pathways of cell surface–receptor signalling, and HSF1-mediated heat shock response regulation, cell cycle regulation were characteristic to LPA.

Assuming that the tumourigenesis proceeds along with the AIS–MIA–LPA axis, the key protein networks identified in this study appear to reflect the nature of the cancer cells of these three subtypes. Generally, the following two possible scenarios can be considered: either LPA cells emerge via transformation of MIA cells or from a different cell origin. The former scenario requires LPA cells to have the same gene mutation profiles as MIA cells and transformed LPA cells to possibly not grow so rapidly but instead co-exist with MIA cells. Conversely, the latter scenario instead requires both MIA and LPA cells to have distinctly different gene mutation profiles from one another. Cancer stem cells (CSCs) are known to emerge via the dysfunctional organogenesis of organ stem cells resulting from the loss of gene regulatory control^43^. In this process, new aberrant cells emerge and acquire a self-renewing capability via the neoplastic transformation of CSCs. Such aberrant cells would emerge as a subpopulation of tumour cells owing to genetic intra-tumour heterogeneity, and their rapid growth would disrupt tumour environment and result in predominant cell survival. The orthogonal partial least square-discriminant analysis (OPLS-DA)^44^ performed for mutant proteins identified in the previous study revealed the profound differences in distance between the LPA group and the AIS plus MIA group^11,12^.

The mutational landscape shown in the larger study conducted by Qian et al.^42^ suggests that a majority of patients in their study have an ethnic background different from ours. Our cohort has a higher rate of oncogenic *EGFR* mutations (57% vs 29%) and a lower rate of *KRAS* mutations (0% vs 25%). Moreover, in the study performed by Chen et al.^41^ oncogenic drivers were not detected in up to half of their samples. Although our study sample size is limited and ethnic background is different, we found similarities between two studies of which oncogenic drivers were detected in MIA and LPA (Fig. 7A, B). Besides, we also found that LPA had a higher degree of copy number loss (one or two copy deletion) than MIA (Fig. 7C), which feature was also seen in the genomic study^42^.

In conclusion, our results could identify disease-related protein networks that are possibly associated with distinct early lung adenocarcinomas—AIS, MIA, and LPA. The large genomic studies suggested mutation signature profiling did not vary significantly throughout pre-invasive lung ADCs—AIS and MIA and invasive lung ADCs^41,42^. However, together with our protein network-based profiles and the previous OPLS discriminant analysis of mutant proteomes^11^, it remains disputable that LPA cells emerge via a direct transformation from AIS or MIA, whereas no evidence was attained by the genomic alteration analysis performed in this study. It should be also noted that their studies grouped all 11 subtypes^45^ including abundant papillary and acinar ADCs as invasive lung ADCs. A large-scale genomic alteration study using tissue specimens histologically well-defined as LPA would be needed for further investigation. Network-based investigations regarding tumourigenesis will further provide clinically important information about proteogenomic landscapes in lung adenocarcinoma.

## Methods

### FFPE tissue specimens and sample preparation

Among 974 patients who underwent surgical lung cancer resection at St. Marianna University Hospital between 2000 and 2014, only 674 (69.3%) had tumours that were histologically confirmed adenocarcinomas. The pathological specimens were independently reviewed by two pathologists (H. N. and M. T.) to confirm that they satisfied the 2015 World Health Organization classification criteria of lung tumours (histological criteria)^46^. FFPE tumour tissue blocks from 14 surgical specimens histologically well-judged as AIS, MIA, and LPA were obtained without patient identifiers from the St. Marianna University School of Medicine Hospital. Informed consent was obtained from all participating subjects, and the protocol was approved by the institutional review board of St. Marianna University School of Medicine (approval no. 1461) and was conducted in accordance with the Helsinki Declaration. For tissue microdissection, 10-μm-thick sections from the FFPE tumour blocks were cut and placed on DIRECTOR slides (OncoPlex Diagnostics Inc., Rockville, MD, USA). The sections were deparaffinised and stained with haematoxylin using standard histological methods prior to dissection. Microdissection was performed using a Leica LMD7 microdissection microscope (Leica, Wetzlar, Germany). A total area of 4 mm^2^ with approximately 15,000 tumour cells was directly transferred from the FFPE sections via laser dissection into the cap of a 200-μL low-binding tube. Proteins were extracted and digested with trypsin using LIQUID TISSUE MS Protein Prep kits (OncoPlex Diagnostics Inc.) according to the manufacturer’s protocol^47^. The procedures have been described in detail elsewhere^12,13^.

A total of eight tumour samples (four MIA and four LPA) selected from the specimens used for proteomic analyses, were subjected to NGS. The RECOVER ALL Total Nucleic Acid Isolation kit (Qiagen, Hilden, Germany) was used to isolate genomic DNA from FFPE tumour samples. The DNA concentration and integrity were analysed using the QUBIT-IT dsDNA HS assay (Invitrogen, Carlsbad, CA, USA) and FRAGMENT ANALYSER (Advanced Analytical Technologies, Ankey, IA, USA), respectively.

### Liquid chromatography-tandem MS (LC–MS/MS)-based proteomic analysis

A label-free quantitation approach using spectral counting by LC–MS/MS was adopted for the global proteomic analysis. The digested samples (5 μL for a single run) were analysed in triplicate by LC–MS/MS using a reverse-phase LC system interfaced with a Q EXACTIVE ORBITRAP mass spectrometer (Thermo Fisher Scientific, Waltham, MA, USA) via a nano-electrospray ionisation device (AMR Inc., Tokyo, Japan). LC–MS/MS analysis has been described in detail previously^13^. The expressions of the identified proteins were assessed by spectral count-based protein quantification^48^.

### Tumour sequencing and analysis of genetic alterations

The NGS was performed with the ACTONCO panel. The extracted genomic DNA was amplified by a polymerase chain reaction to enrich the targeting coding exons of the analysed genes. For variant analysis, raw reads were mapped to the hg19 reference genome using the ION TORRENT SUITE version 5.2 software program (Thermo Fisher Scientific). Coverage depth was calculated using TORRENT COVERAGE ANALYSIS PLUG-IN. Single-nucleotide variants and short insertion/deletions were identified using the TORRENT VARIANT CALLER PLUG-IN (version 5.2; Thermo Fisher Scientific). The coverage was down-sampled to 4,000. The VARIANT EFFECT PREDICTOR version 88 software program (European Bioinformatics Institute, Cambridge, UK) was used to annotate variants using datasets from the Catalogue Of Somatic Mutations In Cancer (COSMIC) version 86 resource and the GENOME AGGREGATION database (version 2.0.2; MacArthur Lab, Boston, MA, USA). Variants with coverage ≥ 25 and an allele frequency of ≥ 10% were retained for further analysis. Variants reported in the GENOME AGGREGATION database with a minor allele frequency of > 1% were considered as polymorphisms. The in-house peripheral blood mononuclear cell database of ACT Genomics was used to determine technical errors.

### WGCNA

The similarity among protein expression patterns for all protein pairs was calculated according to their pairwise Pearson’s correlation coefficient, i.e. the similarity between proteins i and j was defined as (1 − *r*_*i,j*_)/2, where *r*_*i,j*_ is the correlation of the protein expression patterns between the two proteins i and j. An adjacency matrix was then computed by increasing the similarity matrix up to the power of 10 to generate a co-expression network with scale-free properties. Subsequently, from the resultant scale-free co-expression network, we generated a topological overlap matrix (TOM) that considers topological similarities between a pair of proteins in the network. Using the dissimilarity according to TOM (1-TOM), we conducted hierarchical clustering to generate a tree that clustered proteins in its branches. Dynamic tree cutting was used to trim the branches to identify protein modules. A protein module was summarised by the top hub protein (referred to as eigen-protein) with the highest connectivity in the module. To identify the protein modules associated with clinical traits, we calculated the correlation coefficients between the eigen-proteins and clinical traits. WGCNA was conducted using a GARUDA PLATFORM GADGET (The Systems Biology Institute, Tokyo, Japan) that implemented the WGCNA pipeline based on the WGCNA R-package^17^.

### Protein–protein interaction network construction

To construct a protein interaction network for a protein module, we used the STRING database (version 11.0)^22^, which accumulates information on protein–protein interactions from various other databases such as IntAct, Reactome, DIP, BioGRID, MINT, KEGG, NCI/Nature PID, The Interactive Fly, and BioCyc. STRING networks were constructed under the criteria for linkage only with experiments, databases, text mining, and co-expression using the default settings, i.e. a medium confidence score of 0.400, a network depth of 0, or 50 interactions. Subsequently, protein networks imported from the STRING database were visualised using CYTOSCAPE version 3.7.2. Functional enrichment results were obtained for canonical pathways considering *p* < 0.05 to be statistically significant.

## Supplementary information

Supplementary Figures.

Supplementary Legends.

## Data Availability

The unfiltered MS datasets generated and analysed in this study have been deposited in the PRIDE archive (https://www.ebi.ac.uk/pride/archive/) via the PRIDE partner repository and jPOST with the dataset identifiers PXD017334 and JPST000741, respectively.
