# Supplementary material for: A proteogenomic profile of early lung adenocarcinomas by protein co-expression network and genomic alteration analysis
Source: Sci Rep. 2020 Aug 12;10:13604. doi: 10.1038/s41598-020-70578-x (PMC7423934; doi:10.1038/s41598-020-70578-x)
Supplement: Supplementary file 1 — Supplementary Figures. [file 41598_2020_70578_MOESM1_ESM.pdf]

Supplementary Information File 1

A Proteogenomic Profile of Early Lung Adenocarcinomas Revealed by Protein Co-expression Network and Genomic Alteration Analysis

Toshihide Nishimura*, Haruhiko Nakamura, Kien Thiam Tan, De-Wei Zhuo, Kiyonaga Fujii, Hirotaka Koizumi, Saeko Naruki, Masayuki Takagi, Naoki Furuya, Yasufumi Kato, Shu-Jen Chen, Harubumi Kato and Hisashi Saji

Supplementary Figure S1.

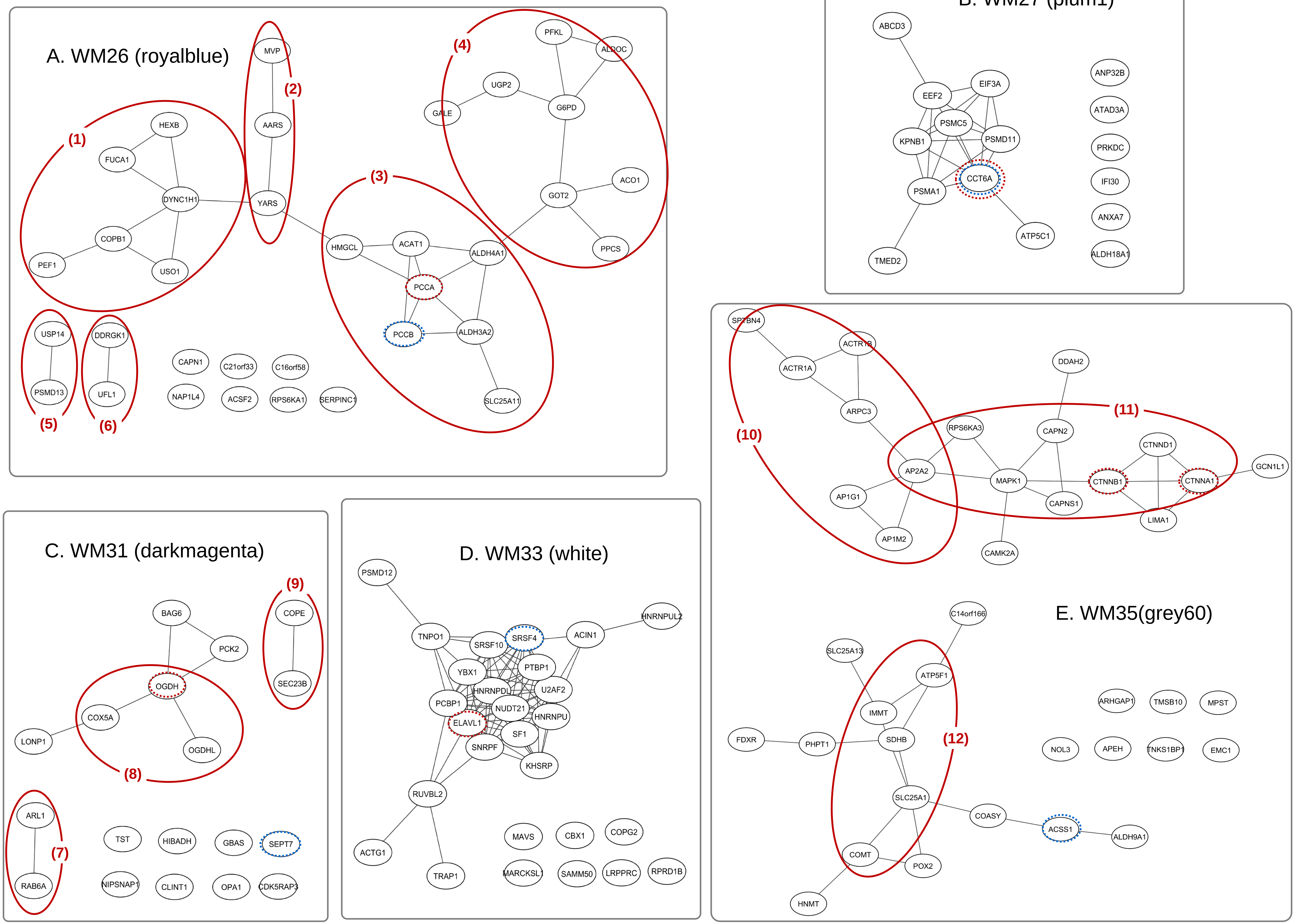

Supplementary Figure S2.

Subtype	WGCNA Molule ID	Biological Process (GO) #term ID	Term description	Observed gene count	False discovery rate	Ractome Pathways #term ID	Term description	Observed gene count	False discovery rate
Lepidic predominant invasive adenocarcinoma (LPA)	WM32 (Dark-magenta)	GO:0009205	purine ribonucleoside triphosphate metabolic process	5	0.00092	HSA-199991	Membrane Trafficking	4	0.0334
		GO:0046034	ATP metabolic process	4	0.0034	HSA-6811440	Retrograde transport at the Trans-Golgi-Network	2	0.0334
		GO:0009150	purine ribonucleotide metabolic process	5	0.0035	HSA-6811442	Intra-Golgi and retrograde Golgi-to-ER traffic	3	0.0334
		GO:0006754	ATP biosynthetic process	3	0.0036	HSA-71291	Metabolism of amino acids and derivatives	3	0.0351
		GO:0009060	aerobic respiration	3	0.0036	HSA-8856688	Golgi-to-ER retrograde transport	2	0.049
		GO:0009167	purine ribonucleoside monophosphate metabolic process	4	0.0036				
		GO:0009168	purine ribonucleoside monophosphate biosynthetic process	3	0.0036				
		GO:0044281	small molecule metabolic process	8	0.0036				
		GO:0048193	Golgi vesicle transport	4	0.0048				
		GO:0000002	mitochondrial genome maintenance	2	0.0057				
	WM27 (Plum1)	N/A	N/A			HSA-382556	ABC-family proteins mediated transport	4	0.00029
						HSA-162909	Host Interactions of HIV factors	4	0.0003
						HSA-109581	Apoptosis	4	0.00061
						HSA-1169091	Activation of NF-kappaB in B cells	3	0.00061
						HSA-1234176	Oxygen-dependent proline hydroxylation of Hypoxia-inducible Factor Alpha	3	0.00061
						HSA-1236974	ER-Phagosome pathway	3	0.00061
						HSA-1236978	Cross-presentation of soluble exogenous antigens (endosomes)	3	0.00061
						HSA-1280215	Cytokine Signaling in Immune system	5	0.00061
						HSA-157118	Signaling by NOTCH	4	0.00061
						HSA-168249	Innate Immune System	6	0.00061
	WM35 (Grey60)	GO:0019886	antigen processing and presentation of exogenous peptide antigen via MHC class II	5	0.0031	HSA-2132295	MHC class II antigen presentation	5	0.0014
		GO:0009987	cellular process	39	0.0034	HSA-164938	Nef-mediates down modulation of cell surface receptors by recruiting them to clathrin adapters	3	0.0019
		GO:0009165	nucleotide biosynthetic process	6	0.0051	HSA-418990	Adherens junctions interactions	3	0.0026
		GO:0042133	neurotransmitter metabolic process	4	0.0054	HSA-442742	CREB phosphorylation through the activation of Ras	3	0.0026
		GO:0009152	purine ribonucleotide biosynthetic process	5	0.006	HSA-5218920	VEGFR2 mediated vascular permeability	3	0.0026
		GO:0050690	regulation of defense response to virus by virus	3	0.006	HSA-373760	L1CAM interactions	4	0.0035
		GO:0051493	regulation of cytoskeleton organization	7	0.006	HSA-437239	Recycling pathway of L1	3	0.0035
		GO:0006839	mitochondrial transport	5	0.008	HSA-444257	RSK activation	2	0.0035
		GO:0009063	cellular amino acid catabolic process	4	0.008	HSA-1643685	Disease	9	0.0037
		GO:0071681	cellular response to indole-3-methanol	2	0.008	HSA-164940	Nef mediated downregulation of MHC class I complex cell surface expression	2	0.0049
	WM33 (White)	GO:0000375	RNA splicing, via transesterification reactions	12	1.41E-12	HSA-72163	mRNA Splicing - Major Pathway	11	1.44E-13
		GO:0008380	RNA splicing	13	1.41E-12	HSA-8953854	Metabolism of RNA	14	2.01E-12
		GO:0006397	mRNA processing	13	3.21E-12	HSA-109688	Cleavage of Growing Transcript in the Termination Region	4	0.00012
		GO:1903311	regulation of mRNA metabolic process	11	3.21E-12	HSA-450531	Regulation of mRNA stability by proteins that bind AU-rich elements	4	0.00021
		GO:0000398	mRNA splicing, via spliceosome	11	1.43E-11	HSA-72187	mRNA 3'-end processing	3	0.0023
		GO:1903312	negative regulation of mRNA metabolic process	8	1.79E-11	HSA-75067	Processing of Capped Intronless Pre-mRNA	2	0.0217
		GO:0006396	RNA processing	14	8.47E-11	HSA-73857	RNA Polymerase II Transcription	7	0.029
		GO:0048025	negative regulation of mRNA splicing, via spliceosome	5	1.96E-08				
		GO:0050684	regulation of mRNA processing	7	1.96E-08				
		GO:0048024	regulation of mRNA splicing, via spliceosome	6	8.58E-08				
	**WM26 (Royal-blue)**	GO:0044281	small molecule metabolic process	20	1.61E-09	HSA-71387	Metabolism of carbohydrates	8	0.00000391
		GO:0019752	carboxylic acid metabolic process	15	3.71E-09	HSA-1430728	Metabolism	15	0.0000678
		GO:0044282	small molecule catabolic process	10	0.000000292	HSA-6798695	Neutrophil degranulation	8	0.0000922
		GO:0006732	coenzyme metabolic process	9	0.000000474	HSA-70326	Glucose metabolism	4	0.00082
		GO:0051186	cofactor metabolic process	10	0.00000118	HSA-70263	Gluconeogenesis	3	0.0013
		GO:0019318	hexose metabolic process	7	0.0000018	HSA-168249	Innate Immune System	9	0.0014
		GO:0005975	carbohydrate metabolic process	9	0.0000108	HSA-71032	Propionyl-CoA catabolism	2	0.0014
		GO:0032787	monocarboxylic acid metabolic process	9	0.000014	HSA-77289	Mitochondrial Fatty Acid Beta-Oxidation	3	0.0014
		GO:0043312	neutrophil degranulation	9	0.0000147	HSA-3371599	Defective HLCS causes multiple carboxylase deficiency	2	0.0022
		GO:0046395	carboxylic acid catabolic process	7	0.0000155	HSA-77111	Synthesis of Ketone Bodies	2	0.0024
