# Supplementary material for: A proteogenomic profile of early lung adenocarcinomas by protein co-expression network and genomic alteration analysis
Source: Sci Rep. 2020 Aug 12;10:13604. doi: 10.1038/s41598-020-70578-x (PMC7423934; doi:10.1038/s41598-020-70578-x)
Supplement: Supplementary file 2 — Supplementary Legends. [file 41598_2020_70578_MOESM2_ESM.docx]

**Supplementary Information**

Supplementary Figure S1. Rest of protein interaction networks of LPA: A) WM26, B) WM27, C) WM31, D) WM33, and E) WM35 modules. Dotted circle nodes in blue and red represent eigen-proteins and hub proteins, respectively, for each module. Solid red circles with numbers represent subnetworks: (1) vesicle-mediated transport, (2) tRNA aminoacylation for protein translation, (3) carboxylic acid/small molecule metabolic process, (4) carbohydrate metabolic process, (5) protein de-ubiquitination, (6) protein K69-linked ufmylation, (7) retrograde transport at the trans-Golgi-network, (8) aerobic respiration, (9) endoplasmic reticulum to Golgi vesicle-mediated transport, (10) antigen processing and presentation of exogenous peptide antigen via MHC class II, (11) signalling by receptor tyrosine kinases, and (12) mitochondrial dysfunction.

Supplementary Figure S2. Top pathways enriched for the rest of the protein WGCNA modules (WM26, WM27, WM31, WM33, and WM35) obtained for LPA concerning Biological Process (GO) and Reactome pathways.
